# Supplementary material for: Analysis of ergot alkaloid gene expression and ergine levels in different parts of Ipomoea asarifolia
Source: PeerJ. 2025 Jul 15;13:e19692. doi: 10.7717/peerj.19692 (PMC12273697; doi:10.7717/peerj.19692)
Supplement: Supplemental Information 1 — (A) Fold difference of the three reference genes (actG, atp6, and tefA) across different plant parts of I. asarifolia. The Ct value of each reference gene was compared to the average Ct value of the three reference genes in all plant parts to obtain the ΔCt. The fold difference value was calculated using the 2−ΔCt method. Plant organs with different letters are significantly different. (B) Correlation between relative dmaW expression and that of the reference genes in each study part of I. asarifolia (r = 0.841, p = 0.009). Abbreviations: YL, young leaf; ML, mature leaf; S, stem; R, root; FB, flower bud; MF, mature flower; YS, young seed; MS, mature seed. [file peerj-13-19692-s001.docx]

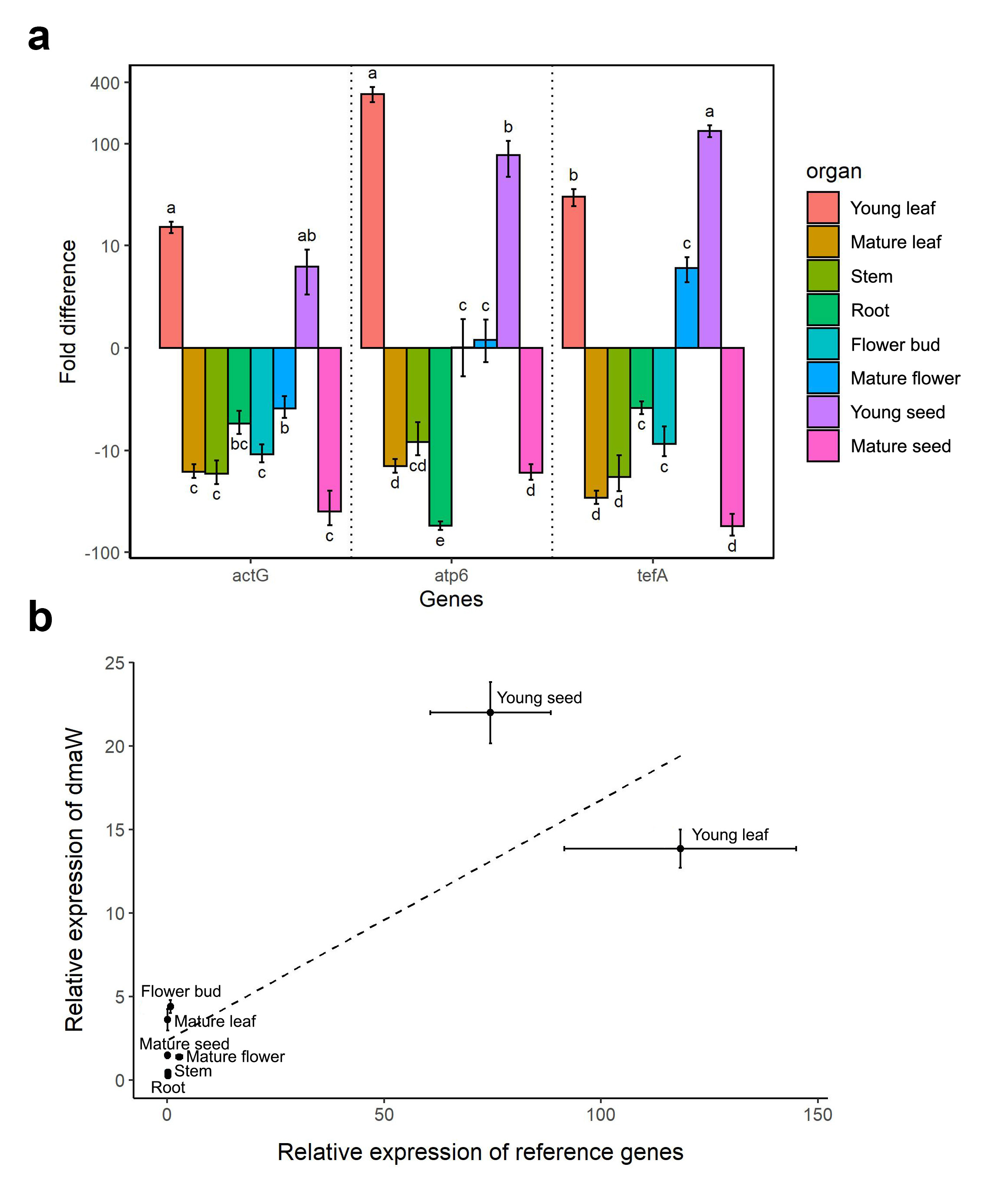


**Figure S1: Fold difference of the reference genes and correlation with *dmaW***

(a) Fold difference of the three reference genes (*actG*, *atp6*, and *tefA*) across different plant parts of *I. asarifolia*. The Ct value of each reference gene was compared to the average Ct value of the three reference genes in all plant parts to obtain the ΔCt. The fold difference value was calculated using the 2^-ΔCt^ method. Plant organs with different letters are significantly different. (b) Correlation between relative *dmaW* expression and that of the reference genes in each study part of *I. asarifolia* (r = 0.841, p = 0.009).
